# Elevated endogenous expression of the dominant negative basic helix-loop-helix protein ID1 correlates with significant centrosome abnormalities in human tumor cells

**DOI:** 10.1186/1471-2121-11-2

**Published:** 2010-01-14

**Authors:** Carolin Manthey, Demissew S Mern, Anja Gutmann, Anne J Zielinski, Corinna Herz, Silke Lassmann, Jens Hasskarl

**Affiliations:** 1 Department of Hematology and Oncology, University Medical Center Freiburg, Freiburg, Germany; 2 Department of Medicine, University Medical Center of Hamburg-Eppendorf, Hamburg, Germany; 3 Helmholtz-University Group Molecular Epidemiology, German Cancer Research Center, Heidelberg, Germany; 4 Department of Cardiology, University Medical Center Freiburg, Freiburg, Germany; 5 Institute of Pathology, University Medical Center Freiburg, Freiburg, Germany

## Abstract

**Background:**

ID proteins are dominant negative inhibitors of basic helix-loop-helix transcription factors that have multiple functions during development and cellular differentiation. Ectopic (over-)expression of ID1 extends the lifespan of primary human epithelial cells. High expression levels of ID1 have been detected in multiple human malignancies, and in some have been correlated with unfavorable clinical prognosis. ID1 protein is localized at the centrosomes and forced (over-)expression of ID1 results in errors during centrosome duplication.

**Results:**

Here we analyzed the steady state expression levels of the four ID-proteins in 18 tumor cell lines and assessed the number of centrosome abnormalities. While expression of ID1, ID2, and ID3 was detected, we failed to detect protein expression of ID4. Expression of ID1 correlated with increased supernumerary centrosomes in most cell lines analyzed.

**Conclusions:**

This is the first report that shows that not only ectopic expression in tissue culture but endogenous levels of ID1 modulate centrosome numbers. Thus, our findings support the hypothesis that ID1 interferes with centrosome homeostasis, most likely contributing to genomic instability and associated tumor aggressiveness.

## Background

The inhibitor of DNA-binding (ID) proteins, ID1-4, are negative regulators of basic Helix-Loop-Helix (bHLH) transcription factors. They lack the basic domain necessary for DNA-binding. By forming DNA-binding incompetent heterodimers with bHLH factors they inhibit transcription of target genes. Various cellular processes are regulated by individual ID-proteins: Inhibition of cellular differentiation by interference with differentiation-specific bHLH and non-bHLH transcription factors [1], extension of cellular life span [2-4], regulation of angiogenesis [5,6] as well as cardiac development [7] and maintenance of the embryonic stem cell phenotype [8]. ID expression is deregulated in many tumors, including cervical cancer [9], melanoma [10], pancreatic cancer [11], squamous cell carcinoma of the esophagus [12] and in thyroid cancer [13]. In some tumors ID-expression is associated with poor clinical prognosis, e.g. in ovarian cancer, in cervical cancer, in prostate cancer, and in breast cancer [9,14-17]. Taken together, these data imply an oncogenic role for ID proteins.

Ectopic expression of ID1 rapidly leads to the accumulation of supernumerary centrosomes in primary human keratinocytes [18], induction of tetraploidy in telomerase-immortalized nasopharyngeal epithelial cells [19], and induction of chromosomal instability through deregulation of APC/Cdh1 in prostate epithelial cells [20]. A fraction of ID1, but not of the other ID proteins, is localized at centrosomal structures. ID1 is the only ID family member that shows a clear association with normal and supernumerary centrosomes throughout the cell cycle [18]. No centrosomal localization can be detected for ID2-4, irrespective of the cell cycle or centrosome duplication status of the cell ([18] and data not shown). Proposed mechanisms of how ID1 can induce centrosomal changes are deregulation of the centrosomal proteasome [21] and stabilization of aurora kinase A [19]. Centrosomes are the microtubule organizing centers (MOC) of the cell and consist of two centrioles surrounded by pericentriolar material containing different coiled-coil proteins, e.g. pericentrin and ninein [22-25]. Centrosome duplication is a critical event during mitosis, as it must only happen once to ensure the formation of a bipolar mitotic spindle and equal segregation of chromosomes during mitosis. Duplication is initiated at the G1-S-phase transition and is controlled by CDK2-Cyclin E/A activity [24]. Furthermore, phosphorylation of pRB seems to be necessary followed by the activity of E2F transcription factors [26]. Centrosome abnormalities are found in neurodegenerative processes as well as in autoimmune diseases, but most frequently they are observed in human malignancies (reviewed in [22,27]). In normal cells centrosome defects lead to G1 arrest of the cell via p53 activation [28]. Tumor cells with mutated p53 lack this mechanism and can still undergo mitosis and thereby accumulate centrosome defects [29]. Furthermore, various cellular and viral oncogenes can induce centrosome abnormalities independent of p53 [18,30-32]. Supernumerary centrosomes lead to the formation of abnormal multipolar mitoses and may ultimately induce aneuploidy [33-35].

Here, we analyzed endogenous ID expression levels in various (tumor) cell lines. By assessing the number of centrosomes we show here that high endogenous ID1 expression, but not that of the other ID proteins, is associated with a higher rate of abnormal centrosomes. This lends further support to the hypothesis that ID1 interferes with centrosomal function and can promote a more aggressive tumor phenotype.

## Results

Ectopic expression of ID1 in primary human cells results in accumulation of supernumerary centrosomes in these cells [18]. High expression levels of the ID-proteins have been observed in various proliferating tissue types [36]. As there are several, partly contradictory reports about the expression levels of the four ID-proteins in primary tumor cells and cell lines, we analyzed 18 established (tumor) cell lines for endogenous ID protein expression levels in proliferating cells.

### ID proteins are differentially expressed in tumor cell lines

**Figure 1:**
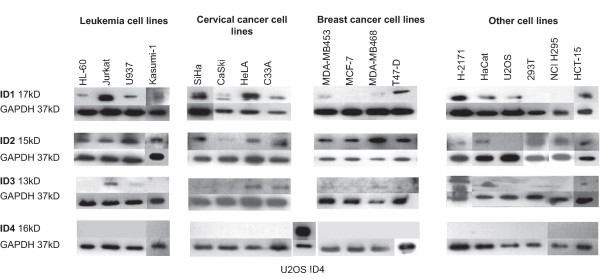
**Endogenous ID expression in different cell lines**. **A) **Expression analysis of ID proteins in leukemic cells. **B) **Expression of ID proteins in cervical cancer cells. **C) **Expression of ID proteins in breast cancer cells. **D) **Expression of ID proteins in other epithelial cells. Cells were grown up to 70% confluence and harvested for protein. Protein extracts (10 μg) were then submitted to western blot analysis. Primary antibodies used were anti-ID1, anti-ID2, anti-ID3 and anti-ID4, GAPDH expression as loading control. Examined cell lines and their characteristics are summarized in Additional File 3.

Protein expression levels of the four ID-proteins were analyzed in proliferating cells using standard Western blotting technique. Cell cycle analysis confirmed similar cell cycle distribution of the different cell lines (Additional File 1). ID protein expression was normalized to GAPDH-expression. Expression of ID1 was readily detectable in all cervical cancer cell lines, in HaCaT, in the colon cancer line HCT-15 as well as in Jurkat (lymphocytic leukemia) (Figure 1A). Intermediate expression of ID1 could be detected in the remaining leukemic cell lines and in two breast cancer cell lines, T47-D and MDA-MB453, whereas MCF-7 and MDA-MB468 only showed very low ID1 expression. ID2 protein expression could be detected in most cell lines (Figure 1B). Only CaSki, a cervical cancer cell line, H-2171 (SCLC), and the osteosarcoma cell line U2OS did not express ID2 in remarkable levels. In contrast higher ID3 expression was only detectable in a small number of cells, i.e. in Jurkat, HaCaT, HeLa, and C33A cells (Figure 1C). ID4 protein was undetectable in all examined cell lines (Figure 1D) confirming earlier reports that ID4 is mainly expressed in neural tissues [37].

### ID-mRNA expression does not correlate with protein expression

**Figure 2:**
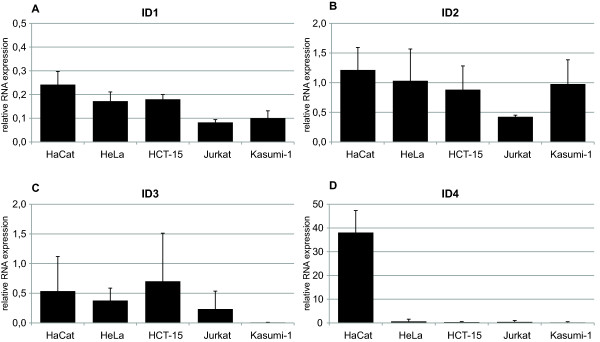
**ID mRNA expression in 5 selected cell lines, assessed by qRT-PCR**; ID-specific primers were used, shown is the mean of at least two independent experiments, bars represent standard deviation; relative values (2^-ΔΔCt^) were calculated using SiHa cells as control cell line, therefore ID mRNA expression was first related to GAPDH mRNA expression in each cell line (ΔCtq), following relation to ID mRNA expression in SiHa cells (ΔΔCt), then submitted to the above mentioned term; A) ID1 mRNA; B) ID2 mRNA; C) ID3 mRNA; D) ID4 mRNA.

To compare mRNA expression of the ID proteins with protein expression, quantitative real time PCR (qRT-PCR) was performed on selected cell lines using ID-specific primer pairs. We observed different ID mRNA expression patterns as compared to ID protein expression patterns (Figure 2). ID1 mRNA expression could be detected at similar levels in all cell lines (Figure 2A). ID2 mRNA expression was readily detectable in all cell lines. Here, only Jurkat seemed to express lower levels of ID2 mRNA, which is in contrast to the relatively high ID2 protein expression in these cells (Figure 2B). ID3 mRNA expression correlated with the ID3 protein expression levels in all cell lines but HCT-15, where the highest mRNA-expression but low protein expression of ID3 was seen (Figure 2C). Most cell lines analyzed showed undetectable ID4 mRNA levels, a finding correlating with non-detectable ID4 protein expression in these cells. Surprisingly, HaCaT cells express high levels of ID4 mRNA, which did not translate into higher ID4 protein levels (Figure 2D).

### Analysis of centrosome number

To address the question whether endogenous ID1 can contribute to accumulation of supernumerary centrosomes, as has been reported for ectopic ID1 expression, the number of centrosomes was determined using immunofluorescence microscopy. A monoclonal antibody against γ-tubulin, a component of the pericentriolar matrix was used to visualize centrosomes. Only cells with one nucleus were analyzed and n > 2 centrosomes per cells were counted as having supernumerary centrosomes. We know from previous experiments that the rate of abnormal centrosomes is approximately 2% in human foreskin keratinocytes. Therefore we assumed a rate of centrosomal abnormalities of > 2% as aberrant.

**Figure 3:**
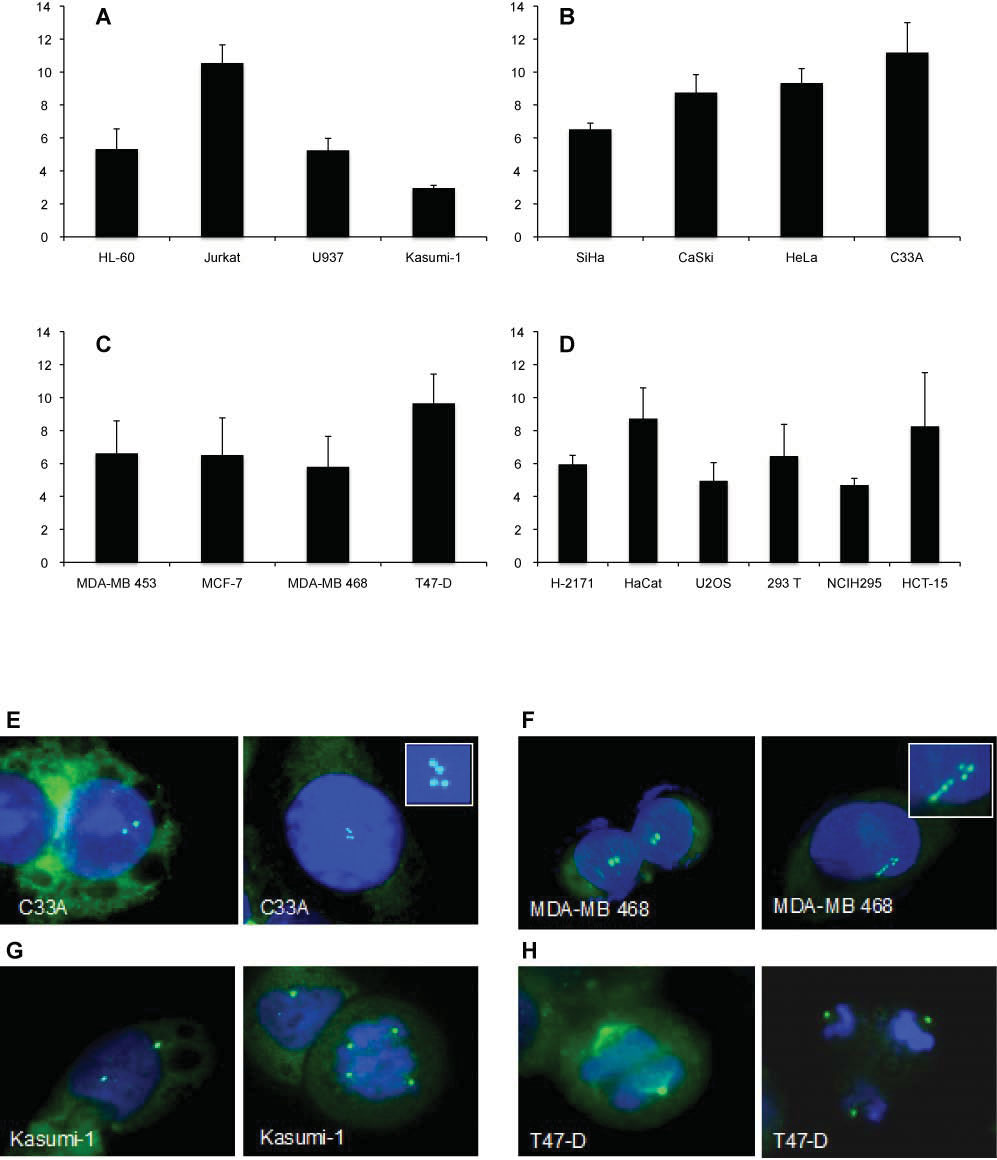
**Assessment of centrosome numbers in established cell lines**. Quantitative analysis of centrosome abnormalities and abnormal mitoses depicted as mean of at least 3 independent experiments, bars represent standard deviation. **A) **Percentage of cells with supernumerary centrosomes in leukemic cells. **B) **Percentage of cells with supernumerary centrosomes in cervical cancer cells. **C) **Percentage of cells with supernumerary centrosomes in breast cancer cells. **D) **Percentage of cells with supernumerary centrosomes in other epithelial cells. **E-H: Immunofluorescence analysis**. Centrosomes were visualized using γ-tubulin antibody (green); nuclei were visualized using Hoechst 33258 (blue); for each group of cell lines representative examples of normal (left picture) and abnormal (right picture) centrosome numbers are shown; **E) **Cell lines with a high rate of centrosomal abnormalities, e.g. C33A **F) **Cell lines with an intermediate rate of centrosomal abnormalities, e.g. MDA-MB468 **G) **Cell lines with a low rate of centrosome abnormalities, e.g. Kasumi-1 **H) **Cell lines with a high number of abnormal centrosomes and mitoses, e.g. T47-D.

Analysis of centrosome number in leukemic cells showed the highest percentage of cells with aberrant centrosomes in Jurkat, followed by HL-60 and U937. Kazumi cells had the lowest percentage of supernumerary centrosomes (Figure 3A &3G). All cervical cancer cells showed a higher percentage of aberrant centrosomes, ranging from 6.5% (SiHa) to 11% (C33A) (Figure 3B &3E). Breast cancer cells showed similar centrosome abnormalities with exception of T47-D (Figure 3C, F &3H). The other tested cell lines showed very heterogeneous centrosome numbers (Figure 3D). The highest numbers of abnormal centrosomes (>9%) were detected in Jurkat, T 47-D, C33A and HeLa cells (summarized in Table 1). An intermediate frequency could be observed in HaCaT, CaSki and HCT-15 with 8.7% (STD ± 1.9), 8.8% (STD ± 1.1) and 8.3% (STD ± 3.3) of supernumerary centrosomes. The remaining cell lines showed a lower number of abnormal centrosomes ranging from 6.6% (± 2.0) in MDA-MB453 to 2.9% (± 0.2) in Kasumi-1 cells (Table 1). Higher ID1 expression correlated with more pronounced centrosomal colocalization (Additional File 2). Centrosome number did not influence the rate of mono- or multipolar mitoses (Table 1). The group of David Pellman recently proposed a direct mechanism by which supernumerary centrosome could induce chromosomal instability: Using long-term live-cell imaging Ganem and colleagues could demonstrate that cells with multiple centrosomes rarely undergo multipolar cell divisions, but routinely undergo bipolar cell divisions. Furthermore, the authors could convincingly show that extra centrosomes alone are sufficient to promote chromosome missegregation during bipolar cell division, and propose this to be the mechanism and the common underlying cause of CIN in human cancer [38].

**Table 1:** Summary of ID expression analysis, centrosome abnormalities and abnormal mitoses.

Cell Line	ID1	ID2	ID3	ID4	% cells with n > 2 centrosomes	% mitoses	% abnormal mitoses
C33A	+	+	(+)	-	11.2 (± 1.8)	6.8 (± 4.5)	1.3 (± 0.9)
JURKAT	+	++	++	-	10.5 (± 1.1)	3.3 (± 0.5)	0.9 (± 0.5)
T47D	(+)	++	-	-	9.7 (± 1.8)	5.5 (± 1.7)	1.2 (± 0.2)
HeLa	+	(+)	(+)	-	9.3 (± 0.9)	6.9 (± 0.2)	0.8 (± 0.2)
CaSki	+	-	-	-	8.8 (± 1.1)	11.0 (± 4.7)	3.8 (± 1.2)
HaCaT	+	(+)	(+)	-	8.7 (± 1.9)	4.2 (± 1.6)	1.7 (± 0.1)
HCT-15	+	+	(+)	-	8.3 (± 3.3)	1.2 (± 1.6)	0.3 (± 0.6)
MDA-MB-453	(+)	(+)	(+)	-	6.6 (± 2.0)	3.2 (± 0.2)	1.2 (± 0.2)
MCF-7	-	(+)	-	-	6.5 (± 2.3)	4.3 (± 1.4)	0.5 (± 0.2)
SiHa	+	(+)	(+)	-	6.5 (± 0.4)	5.3 (± 1.4)	1.5 (± 0.2)
293T	-	(+)	-	-	6.5 (± 1.9)	5.3 (± 0.4)	1.2 (± 0.2)
H-2171	+	-	(+)	-	6.0 (± 0.5)	8.0 (± 1.0)	1.2 (± 0.2)
MDA-MB-468	-	+	(+)	-	5.8 (± 1.8)	2.5 (± 0.7)	0.7 (± 0.7)
HL-60	(+)	++	-	-	5.3 (± 1.2)	6.0 (± 1.0)	0.7 (± 0.2)
U937	(+)	++	-	-	5.2 (± 0.9)	7.3 (± 3.3)	1.1 (± 1.2)
U2OS	(+)	(+)	-	-	4.9 (± 1.1)	3.3 (± 0.5)	1.2 (± 0.7)
NCI-H295	-	(+)	-	-	4.7 (± 0.4)	3.0 (± 0.4)	0.3 (± 0.5)
KASUMI-1	(+)	+	-	-	2.9 (± 0.2)	2.3 (± 1.4)	0.7(± 0.9)

Cell lines are lined up starting with highest rate of centrosomal abnormalities. +: high expression; (+): intermediate expression; -: Low expression or not detectable.

### High ID1 expression correlates with increased centrosomal abnormalities

**Figure 4:**
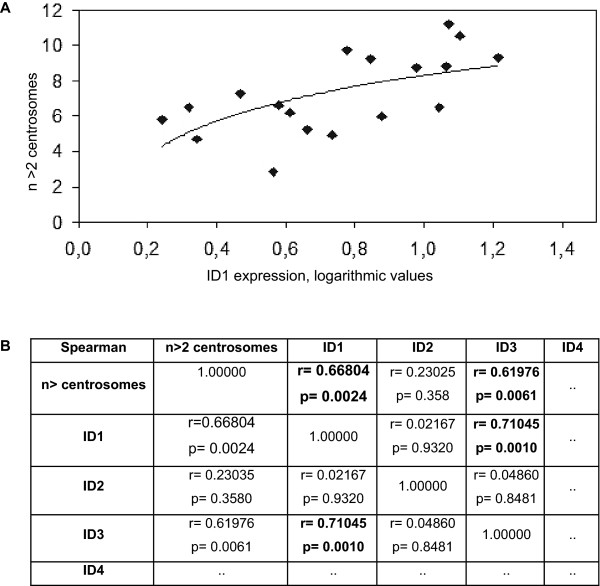
**Statistical data evaluation**. **A) **Logarithmic ID1 expression data compared with number of centrosomal abnormalities; **B) **Spearman's rank correlation coefficient r looking at ID expression and centrosome abnormalities: r values above, p values below; r = 1 shows perfect correlation, r = 0 shows no correlation, 1> r > 0 shows positive correlation, in this case e.g. ID1 and centrosomal abnormalities.

We have previously reported accumulation of supernumerary centrosomes in cells ectopically expressing ID1 [18,21]. No influence of ID2, ID3 or ID4 was detected then. Our findings here were very suggestive that endogenous ID1 expression might also influence centrosomal homeostasis in various cancer cell lines. Comparison of ID1 expression and the number of centrosomal abnormalities showed a clear and significant correlation of high ID1-expression with an increased number of centrosome abnormalities (Figure 4A) (Spearman's rank correlation coefficient p = 0.66804). Surprisingly, not only ID1 expression but also ID3 expression correlated with centrosome abnormalities (Spearman's coefficient p = 0.61976), whereas ID2 and ID4 did not. Similarly, colocalization of ID1 with centrosomes (i.e. with γ-tubulin) was more obvious in leukemic cells expressing higher levels of endogenous ID1 (Additional file 1).

Taken together, elevated endogenous ID1 expression, as seen in diverse cancer cell lines, is associated with an increased number of centrosome abnormalities. Furthermore we have shown here that all ID proteins, except ID4, are expressed in cancer cell lines and that protein expression does not strictly correlate with mRNA expression, most likely due to post-translational modifications and/or ID protein stability and dynamics. Our findings confirm a role of ID proteins in the regulation of centrosome duplication and raise the question of how protein expression of the ID-proteins beyond transcription is regulated.

## Discussion

Although it has been reported that ectopic expression of ID1 but not ID2-4 leads to rapid induction of cells with supernumerary centrosomes [18,19,21] one cannot rule out that this is due to the experimental conditions. We therefore analyzed ID expression in different cell types using immunoblot and real time PCR to correlate endogenous ID expression with centrosome abnormalities. Apart from ID2, which showed a uniform expression pattern in breast cancer and leukemic cells, we did not detect a clear tissue specific expression pattern of the IDs. ID2 is induced in the course of normal granulopoesis [39,40]. ID2 can also be induced by myc through direct promoter activation [41]. Both leukemic cell lines, HL-60 and U937, express myc at high levels, which might explain high ID2 expression in these cells [42]. In addition, ID2 can be induced by HIF-1 and HIF-2 (Hypoxia induced factors), which might contribute to ID2 expression in breast cancer cells [43].

ID3 protein was expressed in a minority of cells, and ID4 protein expression was undetectable in all cell lines analyzed, confirming earlier results [37]. Whether ID4 positively influences tumor growth is still not completely understood. Recent studies revealed that the ID4 gene can be silenced through promoter hypermethylation in various tumors [44-48], suggesting a tumor suppressive role. On the other hand ID4 was identified as an upstream regulator of BRCA1 in breast and ovarian cancer, and more aggressive breast cancer types showed higher ID4 expression [49]. Additionally, activating translocations of ID4 have been detected in some patients and a subset of acute lymphoblastic leukemia [50-52].

ID1 protein expression was readily detectable in most of the cell lines analyzed. Expression was independent of cell cycle distribution or underlying mutations. Since cells were cultured simultaneously under the same conditions, differential induction of ID1 by growth factors from the culture medium is also unlikely. Other factors e.g. proteasomal degradation, RNA stability, microRNAs, posttranscriptional and posttranslational modifications, or gene amplification might regulate ID1 expression. The observed differences between ID protein and mRNA expression imply that ID mRNA or proteins are altered soon after transcription or after translation. ID1, ID2 and ID3 are degraded via the ubiquitin-proteasome complex while ID4 seems to be eliminated through other pathways [53-55]. The half-life of ID proteins, namely ID1 and ID3 is relatively short, depending on the cell type and cell cycle status. It ranges from 30 to 60 minutes [53-55]. Additionally the biological activity of ID2 and ID3 can be altered by phosphorylation, which occurs in late G1 and alters the binding specificity and the stability of these ID proteins [56,57]. By altering the proteasome activity or by accelerating ubiquitination ID protein degradation could be promoted [58]. Intracellular miRNAs or siRNAs might trigger ID mRNA degradation or inhibit translation. Thus, to measure biological activity of the IDs and to further analyze their role in tumor development, protein expression should be determined rather than mRNA expression.

We show here that elevated endogenous ID1 expression levels correlate with the accumulation of abnormal centrosomes. Interestingly, a statistical correlation between ID3 expression and ID1 expression and therefore between ID3 and centrosomal abnormalities was found, which might be due to co-regulation of ID1 and ID3 [59]. Centrosome duplication is orchestrated by cyclins, cyclin-dependent kinases and their inhibitors [60], and loss of the latter results in centriole over duplication [61]. As ID proteins influence members of the cyclin-inhibiting Cip and Kip factors, e.g. p21^Waf1/Cip1 ^and p27^Kip1^, one might assume that ID1 induces centrosome abnormalities by inhibiting p21^Waf1/Cip1^. This seems unlikely, as only ectopic expression of ID1 leads to centrosomal abnormalities, whereas all ID proteins regulate p21^Waf1/Cip1 ^promoter activity [18]. Other (proto)oncogenes such as myc can induce centrosomal abnormalities [21,62,63]. The high-risk human papilloma virus (HPV) E6 and E7 oncoproteins lead to genomic instability and induce abnormal centrosomes [64]. Cervical cancer is most often caused by infection with high-risk HPV [65]. All cervical cancer cell lines analyzed showed high levels of abnormal centrosomes. Interestingly, the highest rate of supernumerary centrosomes was detected in C33A cells, which are HPV negative. Therefore, another mechanism must contribute to accumulation of abnormal centrosomes in these cells, e.g. high expression levels of ID1.

One mechanism that ID1 can deregulate centrosome duplication is by regulating the activity of the centrosomal proteasome. This is in part mediated through interaction of ID1 with S5a, a subunit of the 26S proteasome. ID1 and S5A are both located at centrosomal structures. Ectopic expression of S5a normalizes ID1-induced centrosome abnormalities, and depletion of S5a leads to a similar accumulation of supernumerary centrosomes without tetraploidization [21]. Development of centrosomal alterations and cell aneuploidy has been linked to overexpression of the centrosomal kinase Aurora A [20]. Indeed, it has been shown that ID1 overexpression may lead to stabilization of Aurora A by interaction with the anaphase-promoting complex coactivator Cdh1 [20]. Thus, high levels of ID1 may interact with Cdh1 to stabilize Aurora-A and induce supernumerary centrosomes. The interactions between Aurora kinases and ID1 require further functional in vitro analyses. Aurora-A is differentially regulated and expressed in chromosomal and microsatellite instable colorectal carcinomas and the observed high ID1 expression may contribute to this [66-68]. Recent efforts of therapeutic intervention with small molecule inhibitors of Aurora kinase are ongoing, and the data presented here may provide additional information about the potential therapeutic mechanisms [69-71].

We did not observe a correlation between aneuploidy and increased abnormal centrosomes. Nearly all cell lines analyzed had aneuploid karyotypes (Additional File 3) but not all of them are characterized by high levels of centrosome abnormalities. Even the rate of aneuploidy does not seem to influence the frequency of abnormal centrosomes as MCF-7 and MDA-MB453 reveal only slightly elevated levels of abnormal centrosomes accompanying aneuploidy. Altered p53 function contributes to impaired centrosome function. Only four of the cell lines used (T47-D, MCF-7, U2OS, Kasumi-1) have wild type p53, whereas all other cell lines harbor a mutated or inactivated p53 gene (Additional File 3). We failed to see an influence of p53 status on steady state centrosome numbers. As previously shown, ID1 appears to act independent of p53 as it is able to induce centrosomal abnormalities in p53 deficient as well as in p53 positive cells [18].

## Conclusions

Taken together we show here that not only ectopic but also endogenous ID1 expression can deregulate centrosome duplication, further supporting an oncogenic role for this ID protein. This is in so far of importance, as most knowledge about the function of ID proteins comes from overexpression experiments, in which non-physiologically high expression levels were used to achieve biological effects. Furthermore, when evaluating ID expression in tumors and cell lines, protein levels should be analyzed and correlated with mRNA expression. Our results might imply that protein expression of the IDs is more complex than previously thought and that protein expression and not mRNA-expression should be the main focus of further expression analysis in primary tumors.

## Methods

### Cell culture

Cell lines used were HL-60, Jurkat, U937, Kasumi-1, SiHa, HeLa, CaSki, C33A, MDA-MB468, MDA-MB453, T47-D, MCF-7, HaCaT, U2OS, 293T, NCIH295, HCT-15 and H-2171. L-60, Jurkat, U937, Kasumi-1, MDA-MB453, MCF-7, HCT-15, and H-2171 were maintained in RPMI 1640 medium. NCIH295 cells were grown in RPMI 1640 medium supplemented with 1% insuline-transferrine-selenium-solution. All cervical cancer cell lines (SiHa, HeLa, CaSki, C33A), and T47-D, MDA-MB468, 293T, HaCaT, and U2OS were grown in D-MEM. Medium was supplemented with an antibiotic-antimycotic mixture (streptomycin, penicillin) and 10% (vol/vol) or 15% (NCIH295) heat-inactivated fetal bovine serum. Cells were grown at 37°C, 5% CO2 and 95% vapor-saturated atmosphere.

### Plasmids

Vectors used were pCMV-ID1 (p1121), pCMV-ID2 (p1122), pCMV-ID3 (p1123), pCMV-ID4 (p1124) [2].

### Transfection

U2OS cells were transfected with Fugene 6 (Boehringer Mannheim) according to the manufacturers directions. Cells were harvested for analysis 48 h after transfection.

### Cell harvesting and Western Blotting

Cell lines were grown in 100-mm tissue culture plates to approximately 70% confluence and harvested. After three washes with ice-cold PBS (Gibco) cells were lysed with 100 to 250 μl of lysis buffer (0,5% NP-40 lysis buffer, 50 mM Tris HCl pH 7,4, 150 mM NaCl, and protease and phosphatase inhibitors). Cell lysates were clarified by centrifugation and quantitated by the Bio-Rad DC protein assay system. Samples were then boiled in SDS sample buffer, and equal amounts of proteins were separated by SDS-polyacrylamide gel electrophoresis with 15% acrylamide and transferred to Hybond P membranes (Amersham) for Western blotting analysis. Primary antibodies used included Id1 (C-20), ID2 (C-20), Id3 (H-70), ID4 (H-70) and GAPDH (FL-335) (Santa Cruz, Santa Cruz, CA, USA). Bound proteins were detected with horseradish peroxidase-conjugated secondary antibodies (Santa Cruz, Santa Cruz, CA, USA). After extensive washing of the membrane, fluorescent signal was detected by ECL reagent (Amersham, Braunschweig, Germany), followed by exposure to film (Amersham Hyperfilm ECL, Braunschweig, Germany).

### Image J program

Image J software (Wayne Rasband, National Institute of Mental Health, Bethesda, Maryland, USA) was used to evaluate protein expression. ID expression was normalized to GAPDH expression in each cell line.

### Immunofluorescence

Cells were grown on cover slips to a confluence of 70%, washed with ice-cold PBS and fixed with methanol for 20 minutes at -20°C. After preincubation with normal goat serum for 20 minutes at room temperature to block unspecific protein binding, cells were incubated with primary antibody γ-Tubulin (Sigma, Deisenhofen, Germany) overnight at -4°C. On the next day the cells were incubated for 2 hours with secondary antibody Alexa Fluor 488 (goat-anti-rabbit) (Molecular Probes, Eugene, Oregon, USA) at 37,0°C in a humid chamber. After visualization of nuclei using Hoechst 33258, cover slips were mounted to microscope slides and analyzed using a Zeiss Axioplan confocal microscope. Only mononuclear (i.e. cells containing only one nucleus) cells were analyzed for centrosome number and rate of abnormal mitoses. A minimum of 15 high power fields with at least 20 cells per field was analyzed per cell line. To visualize ID-proteins the above mentioned antibodies were used (1:100 dilution in PBS), then fluorescence labeled secondary antibody Alexa Fluor 568 (Molecular Probes, Eugene, Oregon, USA) was applied (1:200 dilution in PBS).

### RNA isolation

Cells were grown in 100 mm-tissue culture plates to 70% confluence and total RNA was isolated with the RNeasy MicroKit (Qiagen) according to the manufacturers directions. RNA concentration and purity was assessed by spectrophotometry.

### Real-time PCR

Primer pairs (ID1 Forward: 5' TCC AGC ACG TCG ACT ACA 3', ID1 Reverse: 5' GGG TTC CAA CTT CGG ATT CC 3', ID1 Probe: 5' 6-Fam CAG GGA CCT TCA GTT GG MGB 3'; ID2 Forward: 5' CCA CCC TCA ACA CGG ATA TCA 3', ID2 Reverse: 5' CAC AGT GCT TTG CTG TCA TTT G 3', ID2 Probe: 5' 6-Fam TGT CCT TGC AGG CTT CTG MGB 3'; ID3 Forward: 5' CCC CAC CTT CCC ATC CA 3', ID3 Reverse: 5' CAG TGG CAA AAG CTC CTT TTG 3', ID3 Probe: 5' 6-Fam GAC AGC CGA GCT CAC MGB 3'; ID4 Forward: 5' TCC CGC CCA ACA AGA AAG 3', ID4 Reverse: 5' GGT CCA GGA TGT AGT CGA TAA CG 3', ID4 Probe: 5' 6-Fam AGC AAA GTG GAG ATC C MGB 3' were designed using Primer Express (Applied Biosystems). Quantitative reverse transcription-PCR was performed on DNase-treated RNA using the TaqMan Assay (Applied Biosystems) according to the manufacturer's directions. Amplification of target sequences was detected with an ABI 7000 sequence detection system (Applied Biosystems) and analyzed with SDS 2.0 software (Applied Biosystems). The cycle threshold (Ct) was determined as the number of PCR cycles required for a given reaction to reach an arbitrary fluorescence value within the linear amplification range. Relative quantification was performed according to the 2^-ΔΔCt ^method [72], with GAPDH serving as reference and ID1-4 as target genes. First, target and reference CT-values were subtracted from each other (ΔCT) for each sample, followed by subtraction of ΔCT-values of the cell line samples from the reference (SiHa cell line) sample yielding ΔΔCT and final calculation of the fold change by 2^-ΔΔCt^.

### FACS analysis

Cellular DNA content and cell cycle distribution was measured using a Dako Cytomation FACS sorter. After trypsinization cells were pelleted and fixed with 100% ethanol over night. The following day cells were incubated with RNAse A and Propidium iodide for 30 minutes and then subjected to FACS.

### Statistical analysis

Data are provided as mean ± SEM. To determine the correlation between centrosomal abnormalities and ID expression Pearson's correlation coefficient and Spearman's coefficient were calculated following the conversion of ID expression data into logarithmic values. Furthermore r-square was determined to distinguish whether centrosomal abnormalities could be explained by changes of ID expression.

## Authors' contributions

CM did the primary expression analysis and cell analysis and manuscript writing. DSM helped establishing the techniques. AG and AJZ helped with protein expression and cell culture. CH designed the primers and established PCR techniques. SL contributed conceptually and wrote the final manuscript. JH conceptualized the study, helped with experimental procedures, and assessments of the results, wrote and finalized the manuscript. All authors approved the final manuscript.

## Supplementary Material

Additional file 1 **FACS analysis of SiHa, MCF-7, and T47-D cell lines**. DNA content of the cells was evaluated using FACS sorting (Dako Cytomation) after cells were treated with RNAse and Propidium iodide. Representative example of **A**) SiHa, **B**) MCF-7 and **C**) T47-D DNA content (64 corresponds to a single set of chromosomes (G1, G0), 128 to a doubled set of chromosomes (G2), counts in between show cells in S phase. Click here for file

Additional file 2 **Comparison of HL-60 and Jurkat leukemic cell lines**. **A) **Co-Immunofluorescence analysis subcellular localization of ID1 and γ-tubulin; nuclei were visualized using Hoechst 33258 (blue). **B) **Quantitative analysis of cells with n > 2 centrosomes and percent mitotic cells (mean of at least 3 independent experiments, ± standard deviation). **C) **ID1 protein expression in HL-60 and Jurkat cells. Protein extracts (10 μg) were analyzed by immuno blotting using primary antibodies against ID1 (C-20), and GAPDH (loading control). Click here for file

Additional file 3 **Table**. Characteristics of cell lines used. Click here for file
