# Supplementary material for: Elevated endogenous expression of the dominant negative basic helix-loop-helix protein ID1 correlates with significant centrosome abnormalities in human tumor cells
Source: BMC Cell Biol. 2010 Jan 14;11:2. doi: 10.1186/1471-2121-11-2 (PMC2818612; doi:10.1186/1471-2121-11-2)
Supplement: Additional file 3 — Table. Characteristics of cell lines used. [file 1471-2121-11-2-S3.DOC]

**Additional file 3: Characteristics of cell lines used**

| **Cell Line** | **Cell Type** | **Karyotype** | **Known genetic alterations** | **p53 Status** | **Reference** |
| --- | --- | --- | --- | --- | --- |
| **HL-60** | AML | Hypotetraploid; 1,5% Polyploidy |  | inactive | [1] |
| **KASUMI-1** | AML | Hypodiploid | AML1-ETO-gene | wildtype | [2] |
| **JURKAT** | T-cell leukemia | Hypotetraploid; 7,8% Polyploidy |  | inactive | [3] |
| **U937** | NHL | Hypotriploid |  | mutant p53 | [4] |
| **T47D** | Breast Cancer | Hypotriploid; 0,8% Polyploidy |  | wildtype | [5] |
| **MDA-MB-468** | Breast Cancer | Hypertriploid |  | mutant p53 | [6] |
| **MDA-MB-453** | Breast Cancer | Hypotetraploid; 6% Polyploidy |  | mutant p53 | [6] |
| **MCF-7** | Breast Cancer | Hypotetraploid; 8% Polyploidy |  | wildtype | [7] |
| **NCI-H295** | Adrenocortical Cancer | Hypertriploid |  | unknown | [8] |
| **293-T** | Embryonic Kidney | Triploid; 6% Polyploidy |  | mutant p53 | [9] |
| **HCT-15** | CRC | Pseudodiploid; 16% Polyploidy |  | mutant p53 | [10] |
| **H-2171** | SCLC | Hypodiploid; 8% Polyploidy |  | mutant p53 | [11] |
| **U-2 OS** | Osteosarcoma | Hypertriploid | p16 negative | wildtype | [12] |
| **HaCat** | Immortalized keratinocytes | Hypotetraploid |  | mutant p53 | [13] |
| **HeLa** | Cervical carcinoma | Hypertriploid/hypotetraploid; 15% Polyploidy | HPV positive | inactive | [14] |
| **Caski** | Cervical carcinoma | Hypertriploid | HPV positive | inactive | [15] |
| **C33A** | Cervical carcinoma | Pseudodiploid; 8,6% Polyploidy | HPV negative | mutant p53 | [16] |
| **SiHa** | Cervical carcinoma | Hypertriploid; 7,6% Polyploidy | HPV positive | mutant p53 | [17] |

1. Collins, S.J., R.C. Gallo, and R.E. Gallagher, *Continuous growth and differentiation of human myeloid leukaemic cells in suspension culture.* Nature, 1977. **270**(5635): p. 347-9.

2. Asou, H., et al., *Establishment of a human acute myeloid leukemia cell line (Kasumi-1) with 8;21 chromosome translocation.* Blood, 1991. **77**(9): p. 2031-6.

3. Schneider, U., H.U. Schwenk, and G. Bornkamm, *Characterization of EBV-genome negative "null" and "T" cell lines derived from children with acute lymphoblastic leukemia and leukemic transformed non-Hodgkin lymphoma.* Int J Cancer, 1977. **19**(5): p. 621-6.

4. Sundstrom, C. and K. Nilsson, *Establishment and characterization of a human histiocytic lymphoma cell line (U-937).* Int J Cancer, 1976. **17**(5): p. 565-77.

5. Keydar, I., et al., *Establishment and characterization of a cell line of human breast carcinoma origin.* Eur J Cancer, 1979. **15**(5): p. 659-70.

6. Cailleau, R., M. Olive, and Q.V. Cruciger, *Long-term human breast carcinoma cell lines of metastatic origin: preliminary characterization.* In Vitro, 1978. **14**(11): p. 911-5.

7. Soule, H.D., et al., *A human cell line from a pleural effusion derived from a breast carcinoma.* J Natl Cancer Inst, 1973. **51**(5): p. 1409-16.

8. Gazdar, A.F., et al., *Establishment and characterization of a human adrenocortical carcinoma cell line that expresses multiple pathways of steroid biosynthesis.* Cancer Res, 1990. **50**(17): p. 5488-96.

9. Graham, F.L., et al., *Characteristics of a human cell line transformed by DNA from human adenovirus type 5.* J Gen Virol, 1977. **36**(1): p. 59-74.

10. Dexter, D.L., J.A. Barbosa, and P. Calabresi, *N,N-dimethylformamide-induced alteration of cell culture characteristics and loss of tumorigenicity in cultured human colon carcinoma cells.* Cancer Res, 1979. **39**(3): p. 1020-5.

11. Phelps, R.M., et al., *NCI-Navy Medical Oncology Branch cell line data base.* J Cell Biochem Suppl, 1996. **24**: p. 32-91.

12. Heldin, C.H., et al., *A human osteosarcoma cell line secretes a growth factor structurally related to a homodimer of PDGF A-chains.* Nature, 1986. **319**(6053): p. 511-4.

13. Boukamp, P., et al., *Normal keratinization in a spontaneously immortalized aneuploid human keratinocyte cell line.* J Cell Biol, 1988. **106**(3): p. 761-71.

14. Gey GO, Coffman WD Kubicek MT. Cancer Research, 1952, **12**, 264.

15. Pattillo, R.A., et al., *Tumor antigen and human chorionic gonadotropin in CaSki cells: a new epidermoid cervical cancer cell line.* Science, 1977. **196**(4297): p. 1456-8.

16. Auersperg, N., *Long-Term Cultivation of Hypodiploid Human Tumor Cells.* J Natl Cancer Inst, 1964. **32**: p. 135-63.

17. Friedl, F., et al., *Studies on a new human cell line (SiHa) derived from carcinoma of uterus. I. Its establishment and morphology.* Proc Soc Exp Biol Med, 1970. **135**(2): p. 543-5.
